# A Pilot Study: Maternal Undernutrition Programs Energy Metabolism and Alters Metabolic Profile and Morphological Characteristics of Skeletal Muscle in Postnatal Beef Cattle

**DOI:** 10.3390/metabo15030209

**Published:** 2025-03-19

**Authors:** Daichi Nishino, Taketo Haginouchi, Takeshi Shimogiri, Susumu Muroya, Kenji Kawabata, Saki Urasoko, Ichiro Oshima, Shinobu Yasuo, Takafumi Gotoh

**Affiliations:** 1 Graduate School of Bioresource and Bioenvironmental Sciences, Kyushu University, 744 Motooka, Nishi-ku, Fukuoka 819-0395, Japan; nishino.daichi.629@s.kyushu-u.ac.jp (D.N.); syasuo@brs.kyushu-u.ac.jp (S.Y.); 2 Field Science Center for Northern Biosphere, Hokkaido University, Kita 8, Nishi 5, Kita-ku, Sapporo 060-0811, Japan; haginouchi.taketo.b5@elms.hokudai.ac.jp; 3 The United Graduate School of Agricultural Sciences, Kagoshima University, 1-21-24 Korimoto, Kagoshima 890-8580, Japan; simogiri@agri.kagoshima-u.ac.jp (T.S.); muros@vet.kagoshima-u.ac.jp (S.M.); oshima@agri.kagoshima-u.ac.jp (I.O.); 4 Livestock Experiment Station, Kagoshima Prefectural Institute for Agricultural Development, 2440 Kokubuuenodan, Kirishima 899-4461, Japan; kawabata-kenji@pref.kagoshima.lg.jp (K.K.); saki-urasoko@pref.kagoshima.lg.jp (S.U.)

**Keywords:** Wagyu cattle, beef cattle, maternal nutrition, undernutrition, fetal programming, energy metabolism, metabolomics, omics, skeletal muscle, myofiber

## Abstract

**Objectives**: This study investigated the long-term effects of maternal undernutrition on overall muscle metabolism, growth performance, and muscle characteristics in postnatal offspring of Wagyu (Japanese Black) cattle. **Methods**: Wagyu cows were divided into nutrient-adequate (control, CNT; *n* = 4, 120% of requirements) and nutrient-restricted groups (NR; *n* = 4; 60% of requirements), and treated from day 35 of gestation until parturition. Diets were delivered on the basis of crude protein requirements, meeting 100% and 80% of dry matter requirements in CNT and NR groups, respectively. All offspring were provided with the same diet from birth to 300 days of age (d). Longissimus thoracis muscle (LM) samples were collected from the postnatal offspring. **Results**: The NR offspring had lower birth body weight, but their body weight caught up before weaning. These offspring showed enhanced efficiency in nutrient utilization during the post-weaning growth period. Comprehensive analyses of metabolites and transcripts revealed the accumulation of proteinogenic amino acid, asparagine, in NR offspring LM at 300 d, while the abundance of nicotinamide adenine dinucleotide (NADH) and succinate were reduced. These changes were accompanied by decreased gene expression of nicotinamide phosphoribosyltransferase (*NAMPT*), NADH: ubiquinone oxidoreductase subunit A12 (*NDUFA12*), and NADH dehydrogenase subunit 5 (*ND5*), which are essential for mitochondrial energy production. Additionally, NR offspring LM exhibited decreased abundance of neurotransmitter, along with a higher proportion of slow-oxidative myofibers and a lower proportion of fast-oxidative myofibers at 300 d. **Conclusions**: Offspring from nutrient-restricted cows might suppress muscle energy production, primarily in the mitochondria, and conserve energy expenditure for muscle protein synthesis. These findings suggest that maternal undernutrition programs a thrifty metabolism in offspring muscle, with long-term effects.

## 1. Introduction

Maternal nutrition status is one of the extrinsic factors affecting the growth, development, and function of the fetal organ systems [1]. In fetal skeletal muscle, myogenesis primarily drives development, but adipogenesis and fibrogenesis are also initiated during gestation [2]. These tissues are derived from a common pool of mesenchymal progenitor cells in the muscle, and the fate of these progenitor cells is decided during the fetal stage [2]. Thus, nutrition during the fetal stage is a critical factor in determining postnatal growth potential and meat characteristics. Greenwood et al. (2005) reported that severe maternal nutrient restriction reduces postnatal growth [3]. Webb et al. (2019) also found that protein restriction during gestation altered fatty acid composition and total lipid amount in adult offspring muscle [4]. These differences are attributed to the nutritional environment during the fetal stage; however, the mechanism responsible for maintaining these long-term effects remains unclear.

Muscle metabolism influences meat production because muscle growth is a result of protein accretion, which is achieved through complex biological processes including anabolic and catabolic events [5]. In cattle muscle, metabolic and contractile maturations occur during the late fetal stage, while primary and secondary myofibers are formed during the early to mid-fetal stage [6]. We previously found that when the nutrition of Wagyu (Japanese Black) cows was severely restricted throughout the entire gestation period, fetal growth was retarded [7] and fetal muscle metabolism was disrupted at d 260 of gestation [8]. Although many studies have reported that maternal undernutrition impacts the muscle metabolism of ruminant fetuses [9,10,11], few have addressed the long-term effect of maternal nutrient restriction throughout the entire gestation period on the muscle metabolic profile in the offspring of beef cattle postnatally [12].

Conversely, studies using animal models have shown that the skeletal muscle of postnatal offspring from undernourished dams exhibits metabolic alterations, such as reduced mitochondrial energetics, decreased insulin-stimulated glucose uptake, and increased lipid accumulation, resulting in a higher risk of metabolic disease [13,14]. These alterations are considered to result from fetal adaptation through physiological metabolic changes to survive under limited in utero nutrient conditions, thereby programming a thrifty metabolism over the long term [15]. These biological systems might also develop in the skeletal muscle of bovine offspring. Therefore, we hypothesized that maternal undernutrition programs the muscle metabolism of offspring during the fetal stage, which is maintained over the long term and affects the development, functionality, and morphological characteristics of skeletal muscle in postnatal offspring.

The objectives of this study were to determine the long-term effects of maternal nutrient restriction during gestation on overall muscle metabolism, postnatal growth performance, and muscle characteristics in postnatal offspring of Wagyu (Japanese Black) cattle. Specifically, using metabolomics and RNA sequencing, we identified differentially expressed metabolites and transcripts in the longissimus thoracis muscle (LM) of postnatal offspring from Wagyu cows provided with either a control diet or restricted nutrition. This integrative approach revealed that maternal undernutrition changed the fundamental muscle metabolism and functionality of postnatal offspring.

## 2. Materials and Methods

### 2.1. Animals and Experimental Design

**Figure 1:**
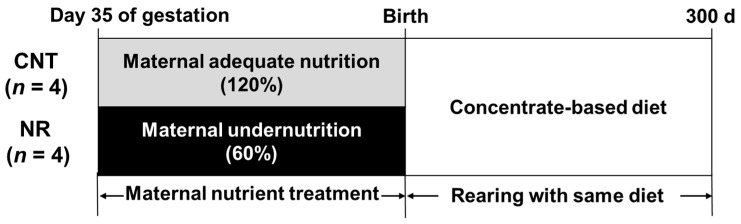
Design of the study. Wagyu cows were provided with adequate nutrition (control, CNT; *n* = 4, 120% of requirements) or restricted nutrition (NR; *n* = 4; 60% of requirements) from d 35 of gestation until parturition. After birth, all offspring were provided with the same diet until 300 d.

The experimental protocols and procedures were reviewed and approved by the Animal Care and Use Committee of Kagoshima University (approval number: A22009). Nutrient requirements, such as crude protein (CP), total digestible nutrients (TDN), and dry matter (DM), were calculated on the basis of animal body weight (BW), in accordance with the Japanese Feeding Standards for Beef Cattle (JFSBC) [16]. Eight multiparous Wagyu (Japanese Black) cows (Kagoshima Prefectural Institute for Agricultural Development, Kirishima, Japan) were fertilized with female-sexed sorted semen of the same sire (Chiehisa bull; National Livestock Breeding Center, Nishishirakawa-gun, Japan). After confirming the pregnancy by ultrasonography at d 35 of gestation, cows were randomly assigned into a nutrient-adequate group (control, CNT; *n* = 4; 120% of JFSBC requirements) or a nutrient-restricted group (NR; *n* = 4; 60% of JFSBC requirements) and the treatments were continued until parturition (Figure 1). The cows in the two groups had comparable BW (CNT: 612.0 kg, NR: 541.8 kg; *p* = 0.15) and body condition score (CNT: 6.5, NR: 5.8; *p* = 0.22). Body condition score refers to the relative amount of subcutaneous body fat in the cow. Two expert evaluators assessed body condition values from one (emaciated) to nine (extremely fat) for six body parts (withers, back, ribs, hooks, pins, and tailhead) through visual inspection and palpation according to the descriptions provided in the Wagyu Registry Association’s Guide [17]. The final score was calculated as the mean of scores from all six anatomical locations. Diets were delivered on the basis of the CP requirements, meeting 100% and 80% of DM requirements in the CNT and NR groups, respectively. Experimental diets consisted of concentrate-based mixed feed (15.3% CP, 69.8% TDN, and 87.6% DM) with wheat straw (3.6% CP, 38.0% TDN, and 85.8% DM) to adjust DM levels, and the diet amount was calculated on the basis of BW. Cows were kept in a drylot, and each cow was fed individually using stanchions to lock in each animal until they completely consumed their feed in the morning (08:30) and afternoon (15:30). CNT offspring were born as the first, third, fifth, and seventh births, while NR offspring corresponded to the second, fourth, sixth, and eighth births. All calves were delivered within the one-month period between 11 October and 4 November 2022, except for the first CNT calf, which was born in August. The offspring calves were provided with colostrum and kept with their mother until 3 days of age (d). After 4 d, all calves were subjected to the same nutritional conditions in a paddock. The calves were provided milk replacer (28.0% CP, 108.0% TDN; Calftop EX Black [Zenrakuren, Tokyo, Japan]) from 4 to 62 d, total mixed ration (TMR) containing hay and dry calf starter (16.8% CP, 72.4% TDN) from 4 to 120 d, and then TMR consisting of hay and concentrate (17.1% CP, 66.2% TDN) from 121 to 300 d. The calves were kept in a drylot and each of them was fed individually using stanchions to lock in each animal in the morning (08:30) and afternoon (15:30). The nutrient intake is shown in Table 1. The calves were weighed at 0, 30, 60, 120, 180, 240, and 300 d (Table 1). All of the cattle were allowed ad libitum access to water and mineralized salt (Cowstone A; Nippon Zenyaku Kogyo, Koriyama, Japan) during the experiment.

### 2.2. Longissimus Thoracis Muscle Sampling

Samples of LM, located at the 12th to 13th thoracic vertebrae, were collected at 75, 180, and 300 d through needle biopsies for histological and transcriptomic analyses. The incision site for needle biopsies was shaved and cleaned with 70% ethanol and diluted povidone–iodine solution. After the administration of a hemostatic agent (5% tranexamic acid; Fujita Pharmaceutical Co., Ltd., Tokyo, Japan), general anesthetic (2% xylazine; Bayer AG, Leverkusen, Germany), and local anesthetic (2% lidocaine hydrochloride; Sandoz Group AG, Basel, Switzerland), the skin was incised, and 2.0 mm wide and 8 cm long LM samples were collected using 15.0 cm sterilized biopsy needles (Merit Medical Systems Inc., South Jordan, UT, USA). Biopsies for histochemical analysis were covered with tissue-embedding medium (Tissue-Tek O.C.T. Compound; Sakura Finetechnical, Tokyo, Japan). All biopsies were immediately frozen in liquid nitrogen and stored at −80 °C until analyses of metabolites, transcripts, and histochemical properties.

### 2.3. Metabolomics and Pathway Analysis

Metabolomic analysis was conducted by capillary electrophoresis time-of-flight mass spectrometry (CE-TOFMS), in accordance with the ω Scan package (Human Metabolome Technologies Inc. [HMT], Tsuruoka, Japan). The methods of Phomvisith et al. [18] were followed with modifications. Briefly, approximately 30–40 mg of LM was mixed with 1500 µL of 50% acetonitrile/Milli-Q water containing 2 µM internal standards (H3304-1002; HMT) and homogenized with zirconia beads (5 mmφ and 3 mmφ) four times at 3500 rpm and 4 °C for 60 s. After centrifugation at 2300× *g* and 4 °C for 5 min, the upper-layer solution was filtered through a Millipore 5-kDa cut-off filter with centrifugation at 9100× *g* and 4 °C for 120 min. The filtrate was lyophilized, reconstituted in Milli-Q water, and analyzed by CE-TOFMS. The spectrometer range was from mass-to-charge ratios (*m*/*z*) of 60 to 900 and 70 to 1050 for cationic and anionic metabolites, respectively. Peak information, including *m*/*z*, peak area, and migration time, was scanned by MasterHands ver.2.19.0.2, automatic integration software (Keio University, Tsuruoka, Japan). Signal peaks were annotated to HMT’s metabolite database based on their *m*/*z* value and migration time. To compare the relative content of the compounds between the CNT and NR groups, the peak areas were normalized to the internal standard amounts and sample weights.

MetaboAnalyst (https://www.metaboanalyst.ca/home.xhtml, accessed on 1 November 2024) was used for the enrichment pathway analysis of differentially expressed metabolites based on the Kyoto Encyclopedia of Genes and Genomes (KEGG) Database (http://www.genome.jp/kegg/pathway.html, accessed on 10 November 2024).

### 2.4. RNA Sequencing and Pathway Analysis

Approximately 100 mg of LM was homogenized in TRIzol reagent (Invitrogen, Carlsbad, CA, USA) and total RNA was extracted in accordance with the manufacturer’s manual (Nippon Gene, Tokyo, Japan) and purified using RNeasy MinElute Cleanup (Qiagen, Hilden, Germany). Total RNA concentration and quality [absorbance (A) ratio at 260 and 280 nm] were quantified using a NanoDrop ND-1000 spectrophotometer (Thermo Fisher Scientific Inc., Waltham, MA, USA). All extractions yielded RNA with an A260:A280 nm ratio greater than 1.9. Messenger RNA (mRNA) was purified from total RNA using poly-T oligo-attached magnetic beads. In accordance with the manufacturer’s manual, synthesized cDNA was paired-end sequenced with NEBNext^®^ Ultra™ II Directional RNA Library Prep Kit (New England Biolabs, Ipswich, MA, USA) using Illumina Platforms (Illumina, San Diego, CA, USA), generating paired-end reads (150 bp in length). The dataset we analyzed was deposited in the DNA Data Bank of Japan (accession numbers: DRR632247–DRR632254)).

Fastp v.0.23.1. [19] was used to check the quality of the sequencing data and filter the sequenced reads. Reads with adaptor sequences, uncertain nucleotides (>10% of either read), and low-quality nucleotides (base quality less than 5, >50% of either read) were removed. The obtained reads were mapped to the bovine reference genome (ncbi_bos_taurus_gcf_002263795_3_ars_ucd2_0) with HISAT2 v.2.0.5 [20] and genes were assembled with StringTie v.1.3.3b [21]. After assembly, transcript abundance was quantified using read counts and normalized as fragments per kilobase of transcript per million mapped reads (FPKM) for each sample. Differential gene expression analysis was performed with DESeq2 [22]. The KEGG pathways enriched with differentially expressed genes (DEG) were estimated by DAVID 2021 (https://david.ncifcrf.gov/, accessed on 10 November 2024) [23].

### 2.5. Muscle and Adipocyte Histochemical Properties and Myofiber Type Composition

Serial and transverse cryosections of LM (8 µm thick) were collected and stained on the basis of their acid-preincubated (pH 4.3) myosin ATPase and reduced nicotinamide adenine dinucleotide dehydrogenase (NADH-DH) activities. Myofiber type was classified as type I, IIA, or IIX, in accordance with the nomenclature of Brooke and Kaiser [24,25], by image analysis using photographs of stained specimens taken with a Biorevo bz-9000 (Keyence, Osaka, Japan) at 100× magnification. Type I myofibers strongly reacted with myosin ATPase after acid preincubation (pH 4.3) and were stained with NADH-DH. Type IIA and IIX myofibers had weak myosin ATPase activity after acid preincubation. Type IIA myofibers reacted with NADH-DH, while type IIX ones did not. Sub-regional myofiber type composition and diameters were assessed on the basis of an average of 300 myofibers. Myofiber diameters were measured as the largest width perpendicular to the myofiber’s long axis. Myofiber type area percentages were estimated using the following equation: (mean of myofiber diameter × 0.5)^2^ × π × (myofiber composition ratio).

Serial and transverse cryosections of LM (8 μm thick) were collected and stained with Oil Red O (FUJIFILM Wako Pure Chemical Corp., Osaka, Japan), which reacted with triglyceride, to detect adipocytes. The cross-sectional area of an average 150 stained adipocytes was analyzed by a Biorevo bz-9000 (Keyence).

### 2.6. Statistical Analyses

All analyses were performed using animal as the experimental unit. To test the effects of diet within the timepoint, the data of birth weight, metabolites, transcripts, myofiber size, and intramuscular adipocyte size were analyzed by a *t*-test. Data for nutrient intake, growth performance, and myofiber type composition were analyzed using two-way ANOVA with adjustments for the repeated measurements. Fixed effects included diet, time, and their interactions, and time served as the repeated measure. Analyses were conducted using the lme4 package in R software (v. 4.4.0), which fits a linear mixed model. Pairwise comparisons between the least squares means of the factor level comparisons were computed using emmeans package in R (v. 4.4.0). To control for multiple comparisons, *p*-values were adjusted using the Bonferroni correction method. For the data on enrichment analysis of the KEGG pathways, adjusted *p*-values were calculated to correct for multiple hypothesis testing (false discovery rate: FDR). Data are shown as the mean ± SEM. Differences were considered statistically significant at *p* ≤ 0.05, while they were considered to show tendencies at *p* ≤ 0.10.

## 3. Results

### 3.1. Growth Performance

No differences in the intake of CP and TDN from milk replacer were found between CNT and NR offspring from 0 to 120 d (*p* > 0.73; Table 1). There were no significant diet × time interactions for the intake of CP and TDN from TMR (*p* > 0.71). While diet did not significantly affect CP and TDN intake from TMR (*p* > 0.43), there were time effects on CP and TDN intake from TMR (*p* < 0.01). The NR offspring had lower birth weights (*p* = 0.03). Significant interactions of diet × time were observed for BW, average daily gain (ADG), and conversion ratio of CP and TDN (*p* < 0.03). Diet had no significant effect on BW, ADG, and conversion ratio of CP and TDN (*p* > 0.34), while time effects were significant for BW, ADG, and conversion ratio of CP and TDN (*p* < 0.03). Maternal undernutrition did not significantly affect offspring BW at 30, 60, 120, and 180 d (*p* > 0.94). At 240 d, NR offspring had greater BW (*p* = 0.03), though this difference was no longer observed at 300 d (*p* = 0.35). There were no differences in ADG between CNT and NR offspring during 0–30 d, 31–60 d, 61–120 d, 121–180 d, and 241–300 d (*p* > 0.69); however, NR offspring had greater ADG during 181–240 d (*p* = 0.04). Finally, there were no differences in the CP and TDN conversion ratio into BW gain between CNT and NR offspring during 0–120 d (*p* > 0.85); however, NR offspring tended to have lower CP and TDN conversion ratio, indicating greater efficient in nutrient utilization (*p* < 0.09).

### 3.2. Metabolomics and Pathway Analysis

In total, 339 metabolite peaks were detected in offspring LM at 300 d and annotated in accordance with the HMT metabolite database (Table S1). In NR offspring LM, there was a higher abundance of pyroglutamine, N^6^,N^6^-dimethyllysine, 11-aminoundecanoic acid, adenosine 3′,5′-diphosphate, and asparagine than in CNT offspring LM (*p* < 0.05; Table 2). Conversely, NR offspring LM had a lower abundance of nicotinamide adenine dinucleotide (NADH), 2-deoxyribonic acid, N-acetylglucosamine 1-phosphate, myo-inositol 2-phosphate, 3-methylcytidine, and allantoic acid (*p* < 0.05). The NR offspring LM tended to have a higher abundance of taurine, methylguanidine, 2-amino-2-methyl-1-propanol, and glycerophosphorylethanolamine (*p* < 0.10). However, there were tendencies for NR offspring LM to have decreased abundance of tyrosine methyl ester, N-acetylaspartylglutamate, fructose 6-phosphate, succinate, gamma-aminobutyric acid (GABA), and glucosaminic acid (*p* < 0.10).

The KEGG pathway enrichment analysis was performed using 21 metabolites that exhibited a significant difference in their abundance between the groups or a tendency for such a difference (Table 3). The pathway of alanine, aspartate, and glutamate metabolism was significantly associated with restricted maternal nutrition, which was enriched by increased asparagine and decreased succinate, N-acetylaspartylglutamate, and GABA (FDR < 0.01). No significant enrichment of other pathways was observed for the differentially expressed metabolites. However, amino acid metabolism and energy metabolism-related pathways, including the tricarboxylic acid (TCA) cycle, were among the top pathways identified. The energy metabolism-related metabolites that showed numerically high differences in abundance (|abundance difference| ≥ 20%) between CNT and NR offspring LM at 300 d are listed in Table S2, regardless of statistical significance.

### 3.3. Transcriptomics and Pathway Analysis

Comprehensive transcriptomics using RNA sequencing analysis revealed 364 upregulated and 263 downregulated DEG in NR offspring LM at 300 d compared with the levels in CNT offspring LM (*p* < 0.05). Genes associated with muscle energy metabolism were identified as DEG (Table 4). Specifically, the gene expression of NADH/ubiquinone oxidoreductase subunit A12 (*NDUFA12*), NADH dehydrogenase subunit 5 (*ND5*), nicotinamide phosphoribosyltransferase (*NAMPT*), pyruvate dehydrogenase E1 subunit alpha 1 (*PDHA1*), and dihydrolipoamide dehydrogenase (*DLD*) were significantly downregulated in NR offspring LM (*p* < 0.03).

There were no KEGG pathways significantly associated with downregulated DEG (Table S3). However, genes related to the cytoskeleton in muscle cells were enriched among the upregulated DEG (FDR < 0.01), including collagen type I alpha 1 chain (*COL1A1*), collagen type VI alpha 1 chain (*COL6A1*), collagen type VI alpha 2 chain (*COL6A2*), and integrin subunit alpha 11 (*ITGA11*; Table 4). Ribosomal-related genes tended to be enriched (FDR = 0.08). Genes related to the cytoskeleton in muscle cells were also enriched in the DEG set, including both upregulated and downregulated DEG (FDR = 0.03).

### 3.4. Muscle and Adipocyte Histochemical Properties

There were no differences in all myofiber type diameters and adipocyte cross-sectional area between CNT and NR offspring LM at 300 d (*p* > 0.32; Table 5). While no diet × time interactions were detected for type IIX myofiber percentage (*p* = 0.91), significant diet × time interactions were observed for the percentages of type I and IIA myofibers (*p* < 0.01; Table 6). There were no differences in type I and IIA percentages between CNT and NR offspring LM at 75 and 180 d (*p* > 0.39). However, NR offspring LM had a lower percentage of type IIA myofibers (*p* = 0.01) and tended to have a higher percentage of type I myofibers (*p* = 0.08) than CNT offspring LM. There were no differences in the estimated area percentages of type IIA and type IIX myofibers at 300 d (*p* > 0.45); however, NR offspring LM had a greater area percentage of type I myofibers than CNT offspring LM (*p* = 0.04; Table S4).

## 4. Discussion

**Figure 2:**
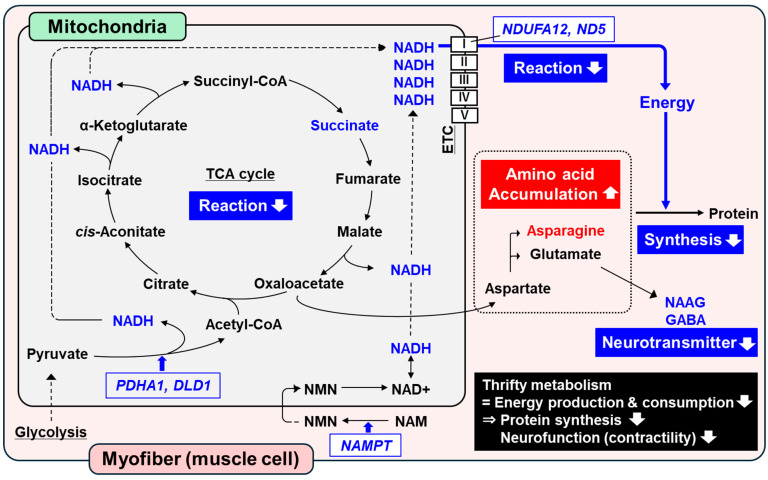
Schematic representation of the effects of maternal undernutrition on energy metabolism of longissimus thoracis muscles (LM) of 300-d-old offspring from cows provided with adequate nutrition (CNT, 120% of requirements) or restricted nutrition (NR, 60% of requirements). Text not enclosed in squares indicates metabolites; red: metabolite with significant accumulation or a tendency toward accumulation (*p* < 0.10), blue: metabolites with significant reductions or a tendency toward reduction (*p* < 0.10), in NR offspring LM; black: metabolites that did not significantly differ in abundance between the groups. Text enclosed in squares and italicized indicates differentially expressed genes (DEG); red: upregulated DEG (*p* < 0.05), blue: downregulated DEG (*p* < 0.05) in NR offspring LM. Proposed reductions in catalytic reaction through the related gene are shown in bold blue arrows. Bold white arrows indicate increased or decreased biological processes. NAD = nicotinamide adenine dinucleotide, NAAG = N-acetylaspartylglutamate, GABA = gamma-aminobutyric acid, NAM = nicotinamide, NMN = nicotinamide mononucleotide, *NDUFA12* = NADH/ubiquinone oxidoreductase subunit A12, *ND5* = NADH dehydrogenase subunit 5, *NAMPT* = nicotinamide phosphoribosyltransferase, *PDHA1* = pyruvate dehydrogenase E1 subunit alpha 1, *DLD* = dihydrolipoamide dehydrogenase. TCA cycle = tricarboxylic acid cycle. I, II, III, IV, and V = complex I, II, III, IV, and V in the electron transport chain (ETC), respectively.

Sustainable and efficient beef production is required to meet the global demand for a nutritious source of protein for the human diet. Studies have reported that maternal undernutrition during gestation impairs offspring growth and productivity [3,4,7]; however, the mechanisms behind the long-term effects remain poorly studied. In the current study, we evaluated the long-term effects of maternal nutrient restriction during gestation on overall skeletal muscle metabolism and morphological characteristics in the skeletal muscle of postnatal offspring. We found that in the LM of offspring from nutrient-restricted cows, proteinogenic amino acid accumulated and TCA cycle-related metabolites decreased. Maternal nutrient restriction reduced the abundance of N-acetylaspartylglutamate and GABA in offspring LM. Additionally, NR offspring LM had a greater proportion of type I myofibers and a lower proportion of type IIA myofibers (Figure 2).

Adequate nutrition throughout gestation is important for beef productivity [26,27]. In the current study, prolonged maternal nutrient restriction decreased offspring birth BW by 10.2%; however, the reduction in BW of NR offspring was no longer observed after 30 d. The NR offspring grew with greater ADG during the post-weaning growth period, resulting in greater BW at 240 d. Although BW at 300 d did not differ between groups, the TDN and CP requirements per unit of BW gain tended to be lower in NR offspring, indicating enhanced nutrient efficiency. Greenwood et al. reported that severe maternal nutrient restriction reduced offspring birth BW, but also described that the cattle growth that was retarded during the fetal stage (with 10.2 kg or 26% lower birth BW) did not catch up with that of well-nourished counterparts at any postnatal age [3]. In the current study, the offspring were provided a concentrate-based diet from pre-weaning to 300 d, whereas in the study of Greenwood et al., cattle were raised with a roughage-based diet from pre-weaning until feedlot entry. Differences in the postnatal environment and ADG could be the reason for the inconsistent findings. Meanwhile, Freetly et al. found that maternal undernutrition during the last two gestations reduced the birth BW of offspring calves; however, the difference was recovered by 2 months [28], similarly to the findings in the current study. Other studies reported that the offspring from undernourished cows could catch up in terms of BW to a level equivalent to that of the controls during the growing period, but maternal nutrient restriction altered the carcass composition. In particular, the carcasses of offspring undernourished in utero had more fat and less lean meat [4,29,30]. Overall, studies suggest that the long-term effects of maternal nutritional restriction on offspring may not be apparent in phenotypic traits such as BW; however, there may be internal changes in muscle metabolism and development.

In the current study, despite all offspring being raised in the same environment for 300 d after birth, 21 differentially expressed metabolites were identified. Maternal undernutrition altered the abundance of the metabolites related to alanine, aspartate, and glutamate metabolism. Specifically, asparagine was increased in NR offspring LM, while succinate, N-acetylaspartylglutamate, and GABA were decreased. Among energy metabolism, this pathway is important for the malate–aspartate shuttle, where aspartate and glutamate play a crucial role in the indirect transfer of NADH from the cytosol into the mitochondrial matrix for oxidation at the mitochondrial electron transport chain (ETC) [31]. Meanwhile, when protein is limited and its biosynthesis is required, aspartate is converted to asparagine by asparagine synthetase [32]. Asparagine is an important proteinogenic amino acid and also promotes protein synthesis through activation of the mammalian target of rapamycin complex 1 (mTORC1) [33]. In the current study, the abundance of asparagine was elevated by 41.1% in NR offspring LM. The anabolic process of proteinogenic amino acids for protein synthesis requires chemical energy, adenosine triphosphate (ATP), mainly produced through the TCA cycle, ETC oxidation, and glycolysis in muscle [5].

NADH, a crucial reducing equivalent, plays essential roles in mitochondrial metabolism. It is primarily produced by the TCA cycle in mitochondria and subsequently oxidized in the ETC, generating ATP [5]. In NR offspring LM, the abundance of NADH was reduced by 77.8%, accompanied by the 39.5% decrease in the abundance of succinate, an intermediate in the TCA cycle. This indicates a suppression of mitochondrial energy production in NR offspring LM, as evidenced by the reduced expression of genes associated with mitochondrial function. Specifically, the gene expression of *NDUFA12* and *ND5*, which encode key subunits of ETC complex I responsible for the oxidation of NADH [34], as well as *NAMPT*, which encodes rate-limiting enzyme mediating NAD+ generation [35], was decreased in NR offspring LM. Furthermore, NADH is also generated in mitochondria during the conversion of pyruvate to acetyl-CoA; however, maternal undernutrition decreased the gene expression of *PDHA1* and *DLD*, which encode subunits of the pyruvate dehydrogenase complex responsible for this process [36]. These findings suggest that maternal undernutrition might suppress chemical energy production in muscle, primarily by impairing mitochondrial functions such as TCA cycle and ETC oxidation.

These alterations of energy metabolism in postnatal offspring were consistent with studies investigating the effect of maternal undernutrition on fetal skeletal muscle. Muroya et al. reported that maternal undernutrition increased the abundance of proteinogenic amino acids, such as glutamine and proline, in bovine fetal LM, accompanied by downregulation of the mRNA expression of genes related to protein synthesis activation [8]. Zhu et al. also demonstrated that maternal nutrient restriction reduced the phosphorylation of mTOR and ribosomal protein, but did not affect calpastatin and ubiquitylated protein content in ovine fetal LM [37]. In addition, Pendleton et al. found that intrauterine growth restriction decreased mitochondrial oxygen consumption and ETC activity in the skeletal muscle of sheep fetuses [38], which was consistent with the findings in a study by Zhao et al. [39]. These studies suggest that maternal nutrient restriction contributes more to the inhibition of protein accretion than to the degradation of existing protein in fetal muscle, suppressing energy consumption and production in muscle mitochondria. Although Fernandes et al. reported long-term effects of protein supplementation during gestation on muscle metabolism in offspring cattle at 22 months [40], few studies have focused on the long-term impact of maternal undernutrition [12]. Studies based on human and animal models have reported significant long-term consequences associated with maternal nutrient restriction. When in utero nutrition is limited, the fetus adapts to that environment through physiological change to survive. This adaptation results in offspring with a thrifty metabolism over the long term, characterized by an increased capacity to store energy fuels rather than burn them [15]. Beauchamp et al. found that maternal nutrient restriction decreased mitochondrial energetics, including respiration ability per mitochondrion, in the muscle of adult offspring mice, accompanied by reduced mitochondrial content and altered muscle characteristics [13]. These studies suggest that maternal undernutrition disrupts metabolism and impairs myofiber development and maturation in offspring LM. In the current study, NR offspring might have adapted to severe maternal nutrient restriction through physiological changes that conserve energy expenditure for protein synthesis in muscle. Reduced mitochondrial energy production and the accumulation of proteinogenic amino acid might be due to the development of a thrifty phenotype originating at the fetal stage.

N-Acetylaspartylglutamate and GABA are responsible for synapse transmission and are well-known markers for neuronal density and viability [41]. Although these metabolites are primarily present in neurons, they are also present and synthesized in nerve terminals [41]. Neural transmission in nerve terminals is important for muscle function because skeletal muscle contractions are neurogenic and are initiated by nerve impulses at the neuromuscular junction [42]. Vliet et al. found that a decrease in the abundance of N-acetylaspartylglutamate is a potential metabolic biomarker of abnormal neurodevelopment related to intrauterine growth restriction [43]. Peerboom and Wierenga reported that GABA is required for neuronal development and function because it is necessary for the migration and proliferation of neuronal precursors, synapse maturation, and neuronal activity and sensitivity [44]. In the current study, maternal undernutrition decreased the abundance of N-acetylaspartylglutamate and GABA by 44.7% and 27.6%, respectively. Additionally, they are synthesized from glutamate [41], which is crucial for amino acid-related energy production. The lack of N-acetylaspartylglutamate and GABA might be due to the alteration of energy metabolism, as stated above, thereby altering neurodevelopment and its function. Therefore, these changes may affect the myofiber contractile functionality.

Skeletal muscles are composed of various myofiber types, classified by their metabolism and contractile properties [45]. Type I myofibers are characterized by oxidative metabolism with higher mitochondrial activity and contract slowly. Meanwhile, type IIX myofibers are classified as glycolytic metabolism and fast contraction, while type IIA myofibers are oxidative-glycolytic and exhibit fast contraction. The distribution and functionality of myofibers are influenced by substrate availability and their fundamental metabolic and contractile abilities, which are established during the last trimester in bovine fetuses [6]. Fahey et al. found that nutrient deficiency during early to mid-gestation increased and reduced slow- and fast-twitch myofibers, respectively, in newborn lamb LM [46]. However, offspring born to nutrient-restricted dams had lower fast oxidative and higher fast glycolytic myofiber percentages at 8 months [47]. Studies investigating matured adult offspring reported that maternal undernutrition during early [48] and mid-gestation [49] did not affect myofiber type composition in LM, consistent with a study by Greenwood and Café, where maternal undernutrition throughout the entire gestation period did not alter myofiber area percentages in offspring LM at 30 months [50]. Other studies targeting late gestation reported that maternal nutrient restriction did not affect myofiber type percentages in the muscle of newborn lambs [51] or 5-month-old offspring [52]. In the current study, maternal nutrient restriction did not alter myofiber composition at 75 and 180 d; however, at 300 d, NR offspring LM exhibited more type I and less IIA myofibers, along with an increased relative area of type I myofibers. The differences among these studies may be attributable to the severity of restriction, timing, and duration of treatment, and muscle development and age of offspring. Matarneh et al. reported that postural muscle requires myofibers characterized by greater energy efficiency, slower contraction kinetics, and higher fatigue resistance [45]. The increase in the percentage of type I myofibers in NR offspring LM at 300 d might be necessary to support heavier BW as they grow. This may indicate an alteration in myofiber functionality, as indicated by the metabolomic analysis. Additionally, the absence of differences in myofiber diameters across all fiber types at 300 d suggests that postnatal diet and environment, rather than prenatal nutrition, may have a sufficient impact on myofiber hypertrophy.

In addition to myofiber formation, the development of adipose and connective tissue is initiated during the prenatal period [6]. Adipogenesis and fibrogenesis are competitive processes with each other because these cellular lineages originate from common fibro-adipogenic progenitor cells, known as mesenchymal stem cells [53]. In the current study, the expression of cytoskeleton-related genes, including collagen and integrin, was increased in NR offspring LM; however, maternal nutrient restriction did not affect adipocyte size. Because adipose tissue has high plasticity [54], the results of the current study might be due to the compensatory growth of adipose tissue in NR offspring LM. Further histochemical and biomolecular investigations are needed to determine whether maternal undernutrition has a long-term effect on the commitment of adipogenesis or fibrogenesis.

In the current study, we set 60% of nutrient requirements as “undernutrition” because, according to JFSBC, this level is considered to represent the minimum amount of nutrition needed to avoid pregnancy complications in pregnant cows [16]. Clarke et al. provided 60% of nutrient requirements to sheep during early to mid-gestation, but ovine fetal size and BW did not differ [55]. By contrast, Vonnahme et al. demonstrated that 50% nutrient restriction decreased fetal BW and surface area of placentomes immediately following a prolonged bout of nutrient restriction from early to mid-gestation in sheep [56]. These studies suggest that moderate restriction and small differences in maternal nutrition levels may not cause significant phenotypic differences in the fetus. Additionally, JFSBC recommends an approximate increase of 25% of the nutrient requirement during the last 2 months of gestation for maintenance of the fetus. We previously reported that fetal BW and tissue/organ weight, such as of muscle and liver, differed between the fetuses from Wagyu cows provided with 60% or 120% of the nutrient requirements [7]. Therefore, in the current study, 120% of the nutrient requirements was set as a control counterpart to 60% of requirements, to investigate the long-term effect of maternal undernutrition on offspring muscle.

Due to the limited animal availability, this study has several limitations that need to be clarified. While we observed significant differences in metabolite abundance, gene expression, and morphological characteristics in muscle, the small sample size may allow the detection of only the most pronounced differences, potentially overlooking subtler yet biologically meaningful variations. Therefore, we emphasize that this research serves as a pilot study investigating the long-term effects of maternal undernutrition on postnatal offspring in beef cattle. To address these limitations, future studies should incorporate larger sample sizes, greater diversity in maternal characteristics (age, parity, breed, and parturition season), and varied levels of maternal nutrient restriction. Such comprehensive investigations would enhance statistical power, improve the detection of subtle effects, and strengthen the robustness and generalizability of findings regarding the impact of maternal nutrient restriction on offspring beef productivity.

## 5. Conclusions

In this study, we found that maternal undernutrition had long-term effects on internal changes in muscle metabolism and functionality, although it did not affect phenotypic traits such as BW over the long term. Maternal undernutrition induced the accumulation of proteinogenic amino acid and reduction in energy production, particularly through decreased NADH production and TCA cycle and ETC oxidation function, in postnatal offspring muscle. Offspring might adapt to the severely limited nutrient environment through physiological changes that reduce mitochondrial energy production and conserve energy expenditure for protein synthesis in muscle. These results might have been due to the thrifty energy metabolism in offspring muscle programmed during the fetal stage. Furthermore, maternal undernutrition decreased metabolites of neurotransmitters, suggesting the long-term impact on the contractile functionality through the alteration of neurodevelopment. Therefore, restricted nutrition during gestation regulates energy metabolism and contractility in offspring muscle over the long term, thereby negatively influencing animal growth and meat productivity.

## Figures and Tables

**Table 1:** Nutrient intake and growth characteristics of offspring Wagyu cattle.

	Treatment ^1^			*p*-Value		
Items	CNT	NR	SEM	Diet	Time	Diet × Time
Nutrient intake, kg						
Milk replacer						
CP ^2^, kg/d	0.14	0.14	0.01	0.73	–	–
TDN ^3^, kg/d	0.53	0.52	0.02	0.73	–	–
Total mixed ration						
CP ^2^, kg/d						
0–120 d	0.27	0.28	0.02	0.45	<0.01	0.71
121–300 d	1.19	1.19	0.04			
TDN ^3^, kg/d						
0–120 d	1.15	1.21	0.07	0.43	<0.01	0.77
121–300 d	4.77	4.81	0.16			
Birth weight, kg	36.1 ^a^	32.4 ^b^	1.2	0.03	–	–
Body weight, kg						
30 d	63.2	55.9	1.2	0.94	<0.01	0.01
60 d	82.3	78.8	2.6			
120 d	140.0	139.2	5.0			
180 d	198.4	205.9	7.0			
240 d	249.3 ^b^	278.3 ^a^	11.1			
300 d	311.1	328.8	12.5			
ADG ^4^, kg/d						
0–30 d	0.90	0.78	0.03	0.34	0.03	0.03
31–60 d	0.64	0.76	0.09			
61–120 d	0.96	1.01	0.05			
121–180 d	0.97	1.11	0.05			
181–240 d	0.85 ^b^	1.21 ^a^	0.16			
241–300 d	1.03	0.84	0.16			
CPCR ^5^, kg CP intake /kg BW gain						
0–120 d	0.47	0.48	0.02	0.86	<0.01	0.03
121–300 d	1.26 ^x^	1.11 ^y^	0.07			
TDNCR ^6^, kg TDN intake /kg BW gain						
0–120 d	1.94	1.99	0.08	0.85	<0.01	0.03
121–300 d	5.07 ^x^	4.47 ^y^	0.31			

^1^ Wagyu cattle offspring from cows provided with adequate nutrition (CNT, 120% of requirements) or restricted nutrition (NR, 60% of requirements). ^2^ CP = crude protein. ^3^ TDN = total digestible nutrients. ^4^ ADG = average daily gain. ^5^ CPCR = CP conversion ratio. ^6^ TDNCR = TDN conversion ratio. ^a,b^ Means at that time point for each myofiber type in CNT vs. NR offspring differ (*p* < 0.05). ^x,y^ Means at that time point for each myofiber type in CNT vs. NR offspring tend to differ (*p* < 0.10).

**Table 2:** Metabolites that differed (*p* < 0.05) or tended to differ (*p* < 0.10) in abundance between the muscles of offspring from nutrient-adequate and nutrient-restricted cows ^1.^

	Treatment ^3^			
Items ^2^	CNT	NR	SEM	*p*-Value
Increased compound in NR offspring LM				
Pyroglutamine	100.0	145.1	8.0	0.01
N^6^,N^6^-Dimethyllysine	100.0	141.3	11.0	0.03
11-Aminoundecanoic acid	100.0	140.7	11.2	0.03
3′,5′-ADP	100.0	203.4	27.6	0.03
Asparagine	100.0	141.4	13.4	0.05
Taurine	100.0	140.3	14.3	0.06
Methylguanidine	100.0	139.0	16.1	0.09
2-Amino-2-methyl-1-propanol	100.0	120.7	8.9	0.10
Glycerophosphorylethanolamine	100.0	171.5	30.4	0.10
Decreased compound in NR offspring LM				
NADH	100.0	22.2	5.7	<0.01
2-Deoxyribonic acid	100.0	72.6	4.6	0.01
N-Acetylglucosamine 1-phosphate	100.0	33.2	12.6	0.01
myo-Inositol 2-phosphate	100.0	68.9	8.9	0.04
3-Methylcytidine	100.0	55.2	12.0	0.04
Allantoic acid	100.0	57.8	13.2	0.05
Tyrosine methyl ester	100.0	69.4	9.5	0.06
N-Acetylaspartylglutamate	100.0	55.3	16.4	0.07
Fructose 6-phosphate	100.0	62.6	12.0	0.07
Succinate	100.0	60.5	13.7	0.07
GABA	100.0	72.4	11.2	0.08
Glucosaminic acid	100.0	63.2	15.2	0.10

^1^ Longissimus thoracis muscles (LM) were collected from 300-day-old offspring from cows provided with adequate nutrition (CNT, 120% of requirements) or restricted nutrition (NR, 60% of requirements). ^2^ Metabolite contents were measured using capillary electrophoresis–time-of-flight mass spectrometry. ^3^ Values in the table represent the mean relative contents as a percentage, with the CNT value set to 100%. NADH = nicotinamide adenine dinucleotide; 3’,5’-ADP = adenosine 3’,5’-diphosphate; GABA = gamma-aminobutyric acid.

**Table 3:** KEGG pathways enriched with metabolites that differed (*p* < 0.05) or tended to differ (*p* < 0.10) in abundance between the muscles of offspring from nutrient-adequate and nutrient-restricted cows ^1^.

			Metabolites ^3^	
Pathway ^2^	*p*-Value	FDR	Increased in NR LM	Decreased in NR LM
Alanine, aspartate and glutamate metabolism	<0.01	<0.01	Asparagine	Succinate, N-Acetylaspartylglutamate, GABA
Butanoate metabolism	<0.01	0.15		Succinate, GABA
Amino sugar and nucleotide sugar metabolism	0.03	0.76		Fructose 6-phosphate, N-Acetyl-glucosamine 1-phosphate
Taurine and hypotaurine metabolism	0.05	1.00	Taurine	
Starch and sucrose metabolism	0.11	1.00		Fructose 6-phosphate
TCA cycle	0.12	1.00		Succinate
Fructose and mannose metabolism	0.12	1.00		Fructose 6-phosphate
Ether lipid metabolism	0.12	1.00	Glycerylphosphorylethanolamine	
Pantothenate and CoA biosynthesis	0.12	1.00	3′,5′-ADP	
Propanoate metabolism	0.13	1.00		Succinate
Pentose phosphate pathway	0.14	1.00		Fructose 6-phosphate
Glycolysis/Gluconeogenesis	0.16	1.00		Fructose 6-phosphate
Arginine and proline metabolism	0.21	1.00		GABA
Glycerophospholipid metabolism	0.21	1.00	Glycerylphosphorylethanolamine	
Primary bile acid biosynthesis	0.26	1.00	Taurine	
Purine metabolism	0.37	1.00		Allantoic acid

^1^ Longissimus thoracis muscles (LM) were collected from 300-d-old offspring from cows provided with adequate nutrition (CNT, 120% of requirements) or restricted nutrition (NR, 60% of requirements). ^2^ KEGG pathway analysis was performed using MetaboAnalyst 6.0 Pathway Analysis. ^3^ Metabolites expressed at higher (CNT < NR) or lower levels (CNT > NR) in NR offspring LM (*p* < 0.10). TCA cycle = tricarboxylic acid cycle; GABA = gamma-aminobutyric acid; 3′,5′-ADP = adenosine 3′,5′-diphosphate.

**Table 4:** Differentially expressed genes related to muscle energy metabolism or cytoskeletal organization between the muscles of offspring from nutrient-adequate and nutrient-restricted cows ^1.^

Biological Process	Gene Symbol	Gene Name	FC ^2^	*p*-Value
Energy metabolism	*NDUFA12*	NADH:ubiquinone oxidoreductase subunit A12	0.49	<0.01
	*ND5*	NADH dehydrogenase subunit 5	0.78	0.01
	*NAMPT*	nicotinamide phosphoribosyltransferase	0.77	0.01
	*PDHA1*	pyruvate dehydrogenase E1 subunit alpha 1	0.79	0.01
	*DLD*	dihydrolipoamide dehydrogenase	0.80	0.03
Cytoskeletal organization	*COL1A1*	collagen type I alpha 1 chain	1.43	0.02
	*COL6A1*	collagen type VI alpha 1 chain	1.33	0.02
	*COL6A2*	collagen type VI alpha 2 chain	1.35	0.04
	*ITGA11*	integrin subunit alpha 11	1.59	0.01

^1^ Longissimus thoracis muscles (LM) were collected from 300-d-old offspring from cows provided with adequate nutrition (CNT, 120% of requirements) or restricted nutrition (NR, 60% of requirements). Gene expression was analyzed by RNA-sequencing. ^2^ FC = fold change of the NR value vs. the CNT value.

**Table 5:** Myofiber and intramuscular fat size of the muscles of offspring from nutrient-adequate and nutrient-restricted cows ^1.^

	Treatment			
Items ^2^	CNT	NR	SEM	*p*-Value
Myofiber diameter at 300 d, µm				
Type I	37.5	41.1	3.0	0.32
Type ⅡA	49.9	54.3	5.5	0.48
Type ⅡX	64.7	64.5	4.3	0.95
Adipocyte size at 300 d, μm^2^				
Cross sectional area	2365.0	1947.4	306.3	0.35

^1^ Longissimus thoracis muscles (LM) were collected from 300-d-old offspring from cows provided with adequate nutrition (CNT, 120% of requirements) or restricted nutrition (NR, 60% of requirements).^2^ Samples of LM adjacent to the 12th to 13th thoracic vertebrae were obtained by needle biopsy. Cryosections (8-μm thick) were prepared and an average of 300 myofibers and 150 adipocytes were measured. The myofiber diameters were measured as the largest width perpendicular to the long axis of each myofiber.

**Table 6:** Myofiber composition of the longissimus thoracis muscles (LM) in offspring from cows provided with adequate nutrition (CNT, 120% of requirements) or restricted nutrition (NR, 60% of requirements) ^1^.

	75 d			180 d			300 d			*p*-Value		
Items	CNT	NR	SEM	CNT	NR	SEM	CNT	NR	SEM	Diet	Time	Diet × Time
Fiber type, %												
Type I	24.7	23.8	2.2	23.3	21.2	1.6	21.6 ^y^	29.0 ^x^	2.7	0.72	0.38	0.01
Type IIA	26.0	26.7	1.2	24.8	27.9	2.1	30.2 ^a^	24.1 ^b^	1.3	0.77	0.05	<0.01
Type IIX	49.2	49.5	2.9	51.9	50.9	1.9	48.2	46.9	1.8	0.92	0.27	0.91

^1^ Samples of LM adjacent to the 12th to 13th thoracic vertebrae were collected by needle biopsy. Cryosections (8-μm thick) were prepared and an average of 300 myofibers and 150 adipocytes were measured. The myofiber diameters were measured as the largest width perpendicular to the long axis of each myofiber. ^a,b^ Means at that time point for each myofiber type in CNT vs. NR offspring differ (*p* < 0.05). ^x,y^ Means at that time point for each myofiber type in CNT vs. NR offspring tend to differ (*p* < 0.10).

## Data Availability

The RNA-sequencing dataset has been deposited in the DNA Data Bank of Japan (accession numbers: DRR632247–DRR632254). Data will be made available on request.

## Supplementary Materials

The following supporting information can be downloaded at: https://www.mdpi.com/article/10.3390/metabo15030209/s1, Table S1: All metabolites detected in the longissimus thoracis muscles of 300-d-old offspring from cows provided with adequate nutrition (CNT, 120% of requirements) or restricted nutrition (NR, 60% of requirements), Table S2: Energy metabolism-related metabolites that showed high differences in abundance (|abundance difference| > 20%) between the muscles of offspring from nutrient-adequate and nutrient-restricted cows, Table S3: KEGG pathways (FDR < 0.10) enriched with the differentially expressed genes (DEG) between the muscles of offspring from nutrient-adequate and nutrient-restricted cows, Table S4: Estimated myofiber area percentages of the longissimus thoracis muscles (LM) in 300-d-old offspring from cows provided with adequate nutrition (CNT, 120% of requirements) or restricted nutrition (NR, 60% of requirements).
